# The *Burkholderia pseudomallei* Proteins BapA and BapC Are Secreted TTSS3 Effectors and BapB Levels Modulate Expression of BopE

**DOI:** 10.1371/journal.pone.0143916

**Published:** 2015-12-01

**Authors:** Puthayalai Treerat, Priyangi Alwis, Tanya D’Cruze, Meabh Cullinane, Jamunarani Vadivelu, Rodney J. Devenish, Mark Prescott, Ben Adler, John D. Boyce

**Affiliations:** 1 Infection and Immunity Program, Monash Biomedicine Discovery Institute, and Australian Research Council Centre of Excellence in Structural and Functional Microbial Genomics, Monash University, Clayton campus, Victoria 3800, Australia; 2 Department of Microbiology, Monash University, Clayton campus, Victoria 3800, Australia; 3 Biochemistry and Molecular Biology, Monash University, Clayton campus, Victoria 3800, Australia; Centre National de la Recherche Scientifique, Aix-Marseille Université, FRANCE

## Abstract

Many Gram-negative pathogens use a type III secretion system (TTSS) for the injection of bacterial effector proteins into host cells. The injected effector proteins play direct roles in modulation of host cell pathways for bacterial benefit. *Burkholderia pseudomallei*, the causative agent of melioidosis, expresses three different TTSSs. One of these systems, the TTSS3, is essential for escape from host endosomes and therefore intracellular survival and replication. Here we have characterized three putative TTSS3 proteins; namely BapA, BapB and BapC. By employing a tetracysteine (TC)-FlAsH^™^ labelling technique to monitor the secretion of TC-tagged fusion proteins, BapA and BapC were shown to be secreted during *in vitro* growth in a TTSS3-dependant manner, suggesting a role as TTSS3 effectors. Furthermore, we constructed *B*. *pseudomallei bapA*, *bapB* and *bapC* mutants and used the well-characterized TTSS3 effector BopE as a marker of secretion to show that BapA, BapB and BapC are not essential for the secretion process. However, BopE transcription and secretion were significantly increased in the *bapB* mutant, suggesting that BapB levels modulate BopE expression. In a BALB/c mouse model of acute melioidosis, the *bapA*, *bapB* and *bapC* mutants showed a minor reduction of *in vivo* fitness. Thus, this study defines BapA and BapC as novel TTSS3 effectors, BapB as a regulator of BopE production, and all three as necessary for full *B*. *pseudomallei in vivo* fitness.

## Introduction

Melioidosis is a disease of humans and animals that causes significant morbidity and mortality in regions where it is endemic, particularly Southeast Asia and Northern Australia [1]. Melioidosis is caused by *Burkholderia pseudomallei*, a Gram-negative, motile, non-spore forming bacillus [2]. *B*. *pseudomallei* is commonly found in tropical environments throughout the world, where infections normally follow inhalation or skin inoculation of the bacteria [3]. The clinical manifestations of melioidosis are diverse, including chronic abscesses, pneumonia and septicemia; these diverse presentations can lead to misdiagnosis [4]. Moreover, infected patients may remain asymptomatic for periods of more than 50 years and recovering patients often suffer recurrent infections [5]. Treatment of *B*. *pseudomallei* infection is difficult due to the intrinsic multidrug resistance of most strains and the ability of the bacteria to survive and replicate within host cells without stimulating the host immune response [3].

Several *B*. *pseudomallei* virulence factors have been characterized, including capsule, lipopolysaccharide (LPS), flagellin, quorum sensing molecules and two type III secretion systems (TTSS) [1,6–9]. TTSSs function by directly injecting bacterial effector proteins into host cells, thus promoting infection by subverting host signal transduction pathways in a manner that benefits bacterial survival [10,11]. TTSSs comprise a basal structure spanning the inner and outer membranes of the bacterium, and an external needle apparatus [12,13] which mediates direct delivery of effector proteins into the host cell [14,15]. Although there are three TTSS loci on the *B*. *pseudomallei* genome, only the first and third clusters (TTSS1 and TTSS3/*bsa*TTSS) have been shown to be involved in virulence [9,16].

*B*. *pseudomallei* can survive and replicate within certain phagocytic and non-phagocytic cells [17,18]. A functional TTSS3 is essential for *B*. *pseudomallei* intracellular survival, as the TTSS3 mediates endosomal escape [19–21]. The TTSS3 is also likely to be important for *B*. *pseudomallei* invasion of some non-phagocytic cells (e.g. A549 cells), but may be dispensable for invasion of other cell types (e.g. HEK293 cells) [22]. As TTSS3 structural mutants are unable to escape from endosomal compartments, they also display a range of downstream phenotypes, including reduced intra- and inter-cellular motility and reduced multinucleated giant cell (MNGC) formation [23]. A functional TTSS3 is also critical for *B*. *pseudomallei* induction of caspase-1 dependent host cell death [24]. Despite the importance of the TTSS3 in the intracellular survival of *B*. *pseudomallei*, the TTSS3 effector proteins are poorly characterized.

The TTSS3 gene cluster encodes a range of TTSS components, including structural proteins, translocators, chaperones, transcriptional regulators and effector proteins [13,25]. The TTSS3 genes *bsaQ* (*BPSS1543*), *bsaU* (*BPSS1539*), *bsaN* (*BPSS1546*), *bicA* (*BPSS1533*), *bsaZ* (*BPSS1534*), *bipB* (*BPSS1532*), *bipD* (*BPSS1529*), *bopA* (*BPSS1524*), *bopB* (*BPSS1514*), *bopE* (*BPSS1525*), *bapA* (*BPSS1528*) and *bapC* (*BPSS1526*), have been characterized to some extent [13,18,26–30]. However, to date, few putative TTSS3 effectors have been confirmed as secreted by wild-type *B*. *pseudomallei* in a TTSS3-dependent manner [20,22,27,31]. BopE was the first component confirmed as a TTSS-secreted effector [27]. BopE is a guanine nucleotide exchange factor that activates the host cell molecules Cdc42 and Rac1. Activation of these molecules leads to host cell actin rearrangement and membrane ruffling [27]. It has been suggested that this activity of BopE is necessary for *B*. *pseudomallei* invasion of HeLa cells, but the TTSS3 does not appear to be essential for invasion of HEK293 cells. A *bopE* mutant was able to escape from the phagosomes of J774.2 murine macrophages, indicating that BopE is not essential for phagosomal escape, and was not attenuated in BALB/c mice [16]. BopA was the second putative effector identified; *bopA* mutant strains show delayed escape from phagosomes, increased susceptibility to killing by LC3-associated phagocytosis and reduced bacterial survival in murine macrophage-like RAW 264.7 cells [20,31]. The third effector protein shown to be secreted *in vitro* in a TTSS3-dependent manner [22] was BopC. Disruption of *bopC* decreased *B*. *pseudomallei* invasion of human lung epithelial A549 cells *in vitro*, suggesting a specific role for BopC in host cell invasion [22]. Recently, a proteomic analysis of two *B*. *pseudomallei* TTSS-hypersecreting mutant strains (*bipD* and *bsaP* mutants) identified 26 proteins as likely TTSS3-dependent effectors [32]. The proteins identified included BopE, BopA and BopC, as well as BapA. Furthermore, BapA, BopA and BprD were confirmed as being secreted in a TTSS3-dependent manner in wild-type *B*. *pseudomallei* strain, using an epitope tagging strategy.

In this study, we characterized the putative TTSS3 proteins, BapA, BapB (*BPSS1527*) and BapC, with respect to their possible functions as bacterial effectors, their roles in controlling expression of other TTSS3 components and their involvement in *B*. *pseudomallei* pathogenesis. A tetracysteine (TC)-FlAsH^™^ labelling technique was used to monitor the secretion of TC-tagged fusion proteins *in vitro*. These analyses confirmed that BapA was secreted in a TTSS3-dependent manner and identified BapC as a novel TTSS3 effector. *B*. *pseudomallei bapA*, *bapB* and *bapC* mutants were constructed and tested for a range of *in vitro* and *in vivo* phenotypes. The essential role of each protein in TTSS3 function was analyzed by using the well-characterized TTSS3 effector BopE as a marker of TTSS secretion activity. All mutants were able to secrete BopE, indicating that BapA, BapB and BapC are not essential for TTSS3 function. However, transcription of *bopE* and secretion of the BopE protein were increased in the *bapB* mutant, indicating that BapB is likely to play a role in modulating *bopE* expression. Finally, all three proteins were shown to have a minor role in *B*. *pseudomallei in vivo* fitness in BALB/c mice.

## Materials and Methods

### Bacterial strains and cell culture

Bacterial strains and plasmids are described in S1 Table. The *B*. *pseudomallei* wild-type strain K96243 [33] (kindly provided by Dr. Brenda Govan, James Cook University, Townsville, Australia) was used as the parent strain for mutagenesis. *Escherichia coli* DH5α was primarily used for growth and amplification of plasmids, and strain S17-1/λ*pir* was used for mobilization of DNA (pDM4 or pBHR1 constructs) into *B*. *pseudomallei* by conjugation [34]. Bacterial culture media were purchased from Oxoid (Hampshire, UK) and solidified by adding 1.5% (wt/vol) agar as required. Antibiotics were purchased from Sigma-Aldrich (St. Louis, MO, USA). Unless indicated otherwise, *E*. *coli* was cultured in Lysogeny broth (LB); ampicillin (Amp; 100 μg/ml), chloramphenicol (Cm; 20 μg/ml), kanamycin (Kan; 50 μg/ml) or tetracycline (Tet; 10 μg/ml) were added when required. *B*. *pseudomallei* was cultured in LB supplemented with gentamicin, (Gen; 8 μg/ml), Cm (50 or 100 μg/ml), Kan (1 mg/ml) or Tet (25 μg/ml) as required. All bacterial cultures were grown at 37°C, with broth cultures shaken at 200 rpm. The murine RAW 264.7 macrophage-like cell line and RAW 264.7 cells stably expressing GFP-LC3 were maintained in antibiotic-free medium as described previously [9,31]. The human respiratory epithelial A549 cell line was obtained from the American Type Culture Collection (Manassas, VA, USA) and maintained at 37°C in 5% CO_2_ in Dulbecco's Modified Eagle Medium DMEM supplemented with 10% (vol/vol) heat-inactivated fetal calf serum (FCS) (GIBCO^®^ Laboratories). All chemical reagents and solvents, unless otherwise stated, were purchased from Merck (Darmstadt, Germany). All restriction endonucleases were purchased from New England Biolabs^®^ (Ipswich, MA, USA).

### Ethics statement

All animal experiments were performed in accordance with the provisions of the “Prevention of Cruelty to Animal Act, 1986”, the “Australian code of practice for the care and use of animals for scientific purposes 7th edition, 2004” and the Monash University Animal Welfare Committee Guidelines and Policies. The experimental procedures were approved by the Monash Animal Research Platform (MARP)-2 Animal Ethics Committee (AEC) of Monash University (AEC number: MARP/2011/067—Pathogenesis in melioidosis).

### Double-crossover, allelic exchange mutagenesis of *bapA*, *bapB* and *bapC*

*B*. *pseudomallei bapA*, *bapB*, and *bapC* mutant strains were generated by double-crossover allelic exchange using the λ*pir*-dependent vector pDM4 which contains the *sacB* gene for counter-selection [35]. Strains and plasmids used are listed in S1 Table. Specific primer pairs (S2 Table) were used to amplify separate sequences upstream and downstream of the target genes, and these were cloned into pDM4. For *bapA* mutagenesis, the primers MC5532 and MC5533 were used to amplify an upstream fragment of *bapA*, and the primers MC5516 and MC5517 were used to amplify a downstream fragment encompassing the entire *bapB* and *bapC* genes. Both fragments were cloned into *Spe*I/*Xba*I-digested pDM4. For *bapB* mutagenesis, the primer pairs JT6156/JT6157 and JT6175/JT6176 were used to amplify the upstream and downstream fragments respectively, and both fragments cloned into *Sph*I/*Spe*I-digested pDM4. For *bapC* mutagenesis, the primer pairs JT6319/JT6320 and JT6179/JT6180 were used to amplify the upstream and downstream fragments, which were then cloned sequentially into *Xma*I/*Sac*I-digested then S*al*I-digested pDM4. The tetracycline resistance gene *tetA*(C), recovered from pUTminiTn*5*Tc [36], was then ligated into the central *Bgl*II site of pDM4 in order to generate the mutagenesis constructs pDM4::*bapA*::*tetA*(C), pDM4::*bapB*::*tetA*(C) and pDM4::*bapC*::*tetA*(C). These constructs were introduced by transformation into the conjugative donor strain *E*. *coli* S17-1/λ*pir*, and then mobilized into *B*. *pseudomallei* by conjugation [9]. Each conjugation reaction was plated onto LB agar containing 8 μg/ml Gen and 25 μg/ml Tet, and plates incubated at 37°C for up to 2 days. Tet resistant transconjugants were patched onto LB agar supplemented with 20% (wt/vol) sucrose in order to select for double-crossover mutants based on *sacB* counter-selection as described previously [37]. Putative double-crossover mutant strains were confirmed by PCR and DNA sequencing (data not shown) and correct strains designated *ΔbapA*, *ΔbapB* and *ΔbapC*.

### Complementation using pBHR1

To confirm that identified phenotypes were due specifically to inactivation of the *bap* genes, each mutant was complemented with the intact gene cloned into the *B*. *pseudomallei* replicating plasmid pBHR1 as described previously [9,28]. Strains and plasmids used are listed in S1 Table and primers in S2 Table. Each of the complementation constructs was then transferred to the appropriate mutant strain by conjugation from *E*. *coli* S17-1/λ*pir*. Tet and Kan resistant, and Cm sensitive colonies were analyzed for the presence of the complementing plasmid using PCR and sequence analysis using the primers flanking the cloning regions These strains were designated *ΔbapA*[*bapA*], *ΔbapB*[*bapB*] and *ΔbapC*[*bapC*]. As a control, the empty plasmid pBHR1 was also transferred into each of the *ΔbapA*, *ΔbapB*, *ΔbapC* strains and the wild-type strain by conjugation. These strains were designated *ΔbapA*[pBHR1], *ΔbapB*[pBHR1], *ΔbapC*[pBHR1] and K96243[pBHR1], respectively.

### Generation of tetracysteine (TC)-tagged *bopE*, *bapA*, *bapB* and *bapC*

Initially a TC-tagged version of *bopE* was constructed in a modified mini-Tn*7* vector (pUC18Tmini-Tn*7*T::*tetA*(C)::*P*_*glmS2*_) that contained a tetracycline gene for selection and the constitutively active *B*. *pseudomallei glmS2* promoter directing transcription of the cloned *bopE* gene. Strains and plasmids are listed in S1 Table and primers in S2 Table. The primers JT6929 and JT6930 were used to amplify an 813 bp fragment containing the full-length *bopE* which was then ligated into *Xma*I/*Spe*I-digested pUC18Tmini-Tn*7*T::*tetA*(C)::*P*_*glmS2*_. The modified TC tag, encoding proline (CCG) and glycine (GGC) as spacers between four cysteine sequences (TGC) and a stop codon (TAA) [38], was generated by annealing the primers JT6931 and JT6932 and then cloning this fragment in-frame at the 3’ end of the *bopE* gene, generating pUC18Tmini-Tn*7*T::*tetA*(C)::*P*_*glmS2*_::*bopE*TC. Initial testing indicated that the expression of TC-tagged BopE from this construct was weak (data not shown), so the TC-tagged *bopE* was transferred into the multi-copy plasmid pBHR1. The primers JT7125 and JT7126 were used to amplify an approximately 1,200 bp fragment containing the *glmS2* promoter, *bopE*, the TC tag, and the two terminator sequences (T_1_ and T_0_) from pUC18Tmini-Tn*7*T::*tetA*(C)::*P*_*glmS2*_::*bopE*TC, and this fragment was then cloned into *Acl*I/*Nco*I-digested pBHR1. The resultant recombinant plasmid was introduced into *E*. *coli* S17-1/λ*pir* and then transferred into the *B*. *pseudomallei* wild-type strain by conjugation. Transconjugants were selected on LB agar containing 8 μg/ml Gen and 1 mg/ml Kan and verified by PCR and nucleotide sequencing (data not shown). One clone containing the correct plasmid was designated *B*. *pseudomallei* [*bopE*TC] and used for further experiments.

Constructs expressing TC-tagged *bapA*, *bapB* and *bapC* were generated by cloning the TC tag into each of the corresponding complementation constructs. In each case, the TC tag was generated by annealing the primers JT7147 and JT7241 prior to ligation into *Acl*I-digested pBHR1::*bapA*, pBHR1::*bapB* or pBHR1::*bapC*. A single correct plasmid representing each construct was then introduced into *E*. *coli* S17-1/λ*pir* and then mobilized into *B*. *pseudomallei* by conjugation. Single pBHR1::*bapA*TC, pBHR1::*bapB*TC and pBHR1::*bapC*TC containing strains were identified and designated K96243 [*bapA*TC], [*bapB*TC] and [*bapC*TC], respectively. In addition, each of the pBHR1::*P*_*glmS2*_::*bopE*TC, pBHR1::*bapA*TC, pBHR1::*bapB*TC and pBHR1::*bapC*TC constructs was transferred into a *B*. *pseudomallei bsaS* mutant [30] by conjugation. Transconjugants were designated *ΔbsaS*[*bopE*TC], *ΔbsaS*[*bapA*TC], *ΔbsaS*[*bapB*TC] and *ΔbsaS*[*bapC*TC], respectively.

### Precipitation of proteins using sodium deoxycholate/trichloroacetic acid (DOC/TCA) and protein sample preparation

For precipitation of proteins, DOC was used, in combination with TCA, to act as a co-precipitant to enhance protein precipitation as described previously [39,40] with some modifications. In brief, culture supernatant samples collected from bacterial cells grown to the required growth phase were filtered through a 0.45-μM syringe filter (Pall Life Science, USA) and subsequently incubated with 0.02% (wt/vol, final concentration) DOC at room temperature for 15 min. Total proteins were precipitated by incubation with 10% (wt/vol, final concentration) TCA overnight at 4°C, followed by centrifugation (10,000 x *g*, 20 min, 4°C). The pellet was washed twice with ice-chilled methanol prior to air-drying to eliminate any methanol residue. Laemmli sample buffer (1X) containing β-mercaptoethanol (BME) was used to resuspend the protein pellet. The samples were then denatured by boiling at 99°C for 10 min and cooled to room temperature before determination of total protein in the samples using the 2-D Quant Kit (GE Healthcare, NSW, Australia) according to the manufacturer’s instructions.

### TC-FlAsH^™^-based fluorescence labelling

TC-tagged proteins were visualized using FlAsH-labelling as described previously [38,41] with some modifications. For visualization of TC-tagged proteins from live cells, *B*. *pseudomallei* was grown to exponential phase, harvested by centrifugation (4,293 x *g*, 5 min, room temperature) and washed with sterile phosphate-buffered saline, pH 7.4 (PBS) prior to resuspension in 50 μl of PBS. Stock FlAsH reagent, from the TC-FlAsH^™^ II In-Cell Tetracysteine Tag Detection Kit (Life Technologies^™^, USA), was added to the bacterial cell suspension to obtain a final concentration of 5 μM prior to incubation at 37°C with shaking at 1,000 rpm, for 1 h. Labeled samples were washed once with PBS to remove unbound FlAsH reagent prior to resuspension in 1X Laemmli sample buffer containing 10 mM (final concentration) dithiothreitol (DTT) and heated at 99°C for 10 min. Labeled proteins were separated by SDS-PAGE, and green fluorescence emission visualized immediately at 520 +/- 10 nm upon excitation at 488 nm. For visualization of secreted TC-tagged proteins *in vitro*, DOC/TCA precipitated supernatant samples were labeled with FlAsH reagent according to the manufacturer’s instructions with the following minor modifications. Precipitated proteins were labeled with the FlAsH reagent (20 μM, final concentration), using 2-mercaptoethanol as the reducing agent, and incubated at 70°C with shaking for 10 min. Samples were cooled to room temperature, polypeptides separated by SDS-PAGE and fluorescently labeled samples visualized as described above.

### Western immunoblotting

Following SDS-PAGE, proteins were transferred to PVDF membranes (Merck Millipore, USA) and the membranes probed by immunoblotting with rabbit anti-BopE_78-261_ antiserum [27] as described previously [30]. Antibody binding was analyzed using Amersham ECL Western Blotting Detection Reagent (GE Healthcare, NSW, Australia), and chemiluminescent signals detected with X-ray film (Kodak, NY, USA). BopE expression in each sample was normalized for sample loading by densitometric analysis of Coomassie Blue stained whole cell lysates and the BopE expression reported as relative to the expression levels in the wild-type strain (fold-change relative to wild-type expression).

### Purification of RNA and quantitative real-time RT-PCR (qRT-PCR)

Total bacterial RNA was extracted using TRIzol^®^ (Life Technologies^™^, USA), and cDNA was produced using reverse transcriptase and random hexamers as described previously [42]. qRT-PCR was conducted with the Mastercycler^®^ ep realplex PCR system (Eppendorf South Pacific, Australia) using FastStart Universal SYBR Green Master (Rox) as the master mix (Roche Diagnostics, Australia). The primer pairs JT7472/JT7473 and JT7474/JT7475 (S2 Table) were designed for the amplification of *bopE* and the internal control *rpoA* (*BPSL3187*), respectively, using the Primer3 primer design software (http://simgene.com/Primer3). These primer pairs amplified PCR products of 119 and 90 bp, respectively. Each qRT-PCR mixture was prepared by addition of 5 μl of 1:10 dilution of cDNA to 15 μl of PCR master mix containing 10 μl of FastStart Universal SYBR Green Master (Rox), 0.2 μl of each primer (100 μM) and 4.6 μl of DEPC-treated nuclease-free water (Ambion). The genomic DNA of *B*. *pseudomallei* wild-type strain K96243 and RT-negative samples were used as positive and negative controls, respectively. The cycling parameters for amplification were as follows: 1 cycle of 95°C for 2 min, 40 cycles of 95°C for 15 sec and 60°C for 1 min. All qRT-PCR experiments were carried out in technical triplicate on biological duplicates.

### *In vivo* competitive growth assays

To determine the ability of each strain to grow *in vivo*, relative growth assays in BALB/c mice were carried out as described previously [9]. In brief, each of the mutant and the wild-type strains was grown to an OD_600_ of 0.2 (corresponding to 2 x 10^8^ CFU/mL). Equal volumes of the mutant and the wild-type cultures were combined and 10 μL of the 10^−2^ dilution of the combined mixture (corresponding to 2 x 10^4^ CFU) was inoculated intranasally into three to five, 6- to 8-week-old, female BALB/c mice. The infection was allowed to proceed for 20 h, and mice were then euthanised in an ethically approved manner (Monash University Animal Ethics Committee approval #SOBS/M/2008/2). Numbers of mutant and wild-type bacteria in input cultures, or in output cultures recovered from mouse spleens, were determined by patching recovered bacteria onto LB plates with or without Tet (25 μg/ml). The competitive index (CI) was defined as the ratio of mutant to wild-type bacteria in the output pool divided by the ratio of mutant to wild-type bacteria in the input pool. The statistical significance of a reduction in CI for each mutant was determined by a one sided z-test [43,44] and the difference in CI between mutant and complemented strains assessed by Mann-Whitney *U* test.

### Statistical analyses

Two-way ANOVA was used to analyze intracellular survival assays, and Student’s unpaired *t*-test was used to compare means between each mutant and the wild-type. A *P* value of less than 0.05 was accepted as indicating a statistically significant difference between samples.

## Results

### The *B*. *pseudomallei* BapA and BapC proteins are secreted by the TTSS3

**Fig 1:**
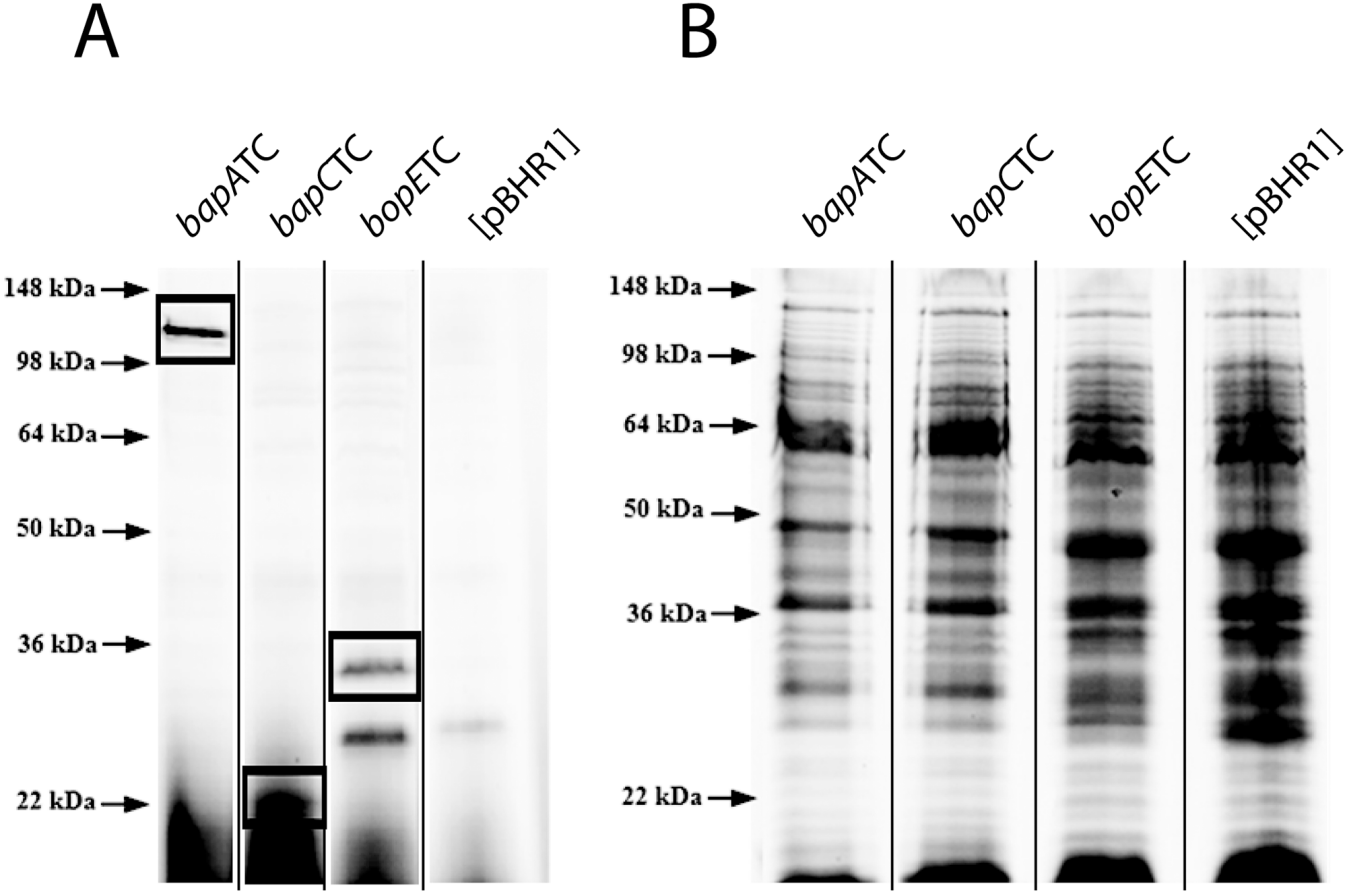
Identification of TC-tagged BapA and BapC by FlAsH labelling of live *B*. *pseudomallei*. Proteins in whole cell lysates of FlAsH-labeled culture samples were separated by SDS-PAGE and visualized by (A) fluorescence or (B) Coomassie Blue staining. Protein bands at or near the expected size of TC-tagged BapA (120 kDa), BapC (23 kDa) and BopE (33 kDa) are indicated by the boxes. The positions of molecular mass markers are shown on the left. Vertical black lines on the gel image indicate that unrelated intervening lanes have been removed.

The *bapA*, *bapB* and *bapC* genes are located within the TTSS3 locus, but the functions of the encoded proteins are unknown. To investigate if these genes might encode secreted TTSS3 effectors, each of the genes was fused with a sequence encoding a TC tag and recombinant C-terminal tagged proteins visualized by labelling with the membrane-permeant 4’,5’-bis(bis1,3,2-dithioarsolan-2-yl) fluorescein (FlAsH) reagent. As a control, the gene encoding the well characterized effector BopE was also fused to the same TC tag. Firstly, to identify whether the FlAsH reagent was able to access and bind to the TC-tagged proteins in the recombinant *B*. *pseudomallei* [*bopE*TC], [*bapA*TC], [*bapB*TC] and [*bapC*TC] strains, mid-exponential phase cultures of these recombinant strains were incubated with the FlAsH compound and then proteins in whole cell lysate samples separated by SDS-PAGE (Fig 1A); as a control for sample loading the total proteins in each sample were visualized by PAGE and Coomassie Blue staining (Fig 1B). A fluorescent signal at 33 kDa, predicted to correspond to TC-tagged BopE, was observed in the *B*. *pseudomallei* [*bopE*TC] sample, but not in the *B*. *pseudomallei* [pBHR1] sample (Fig 1A). In samples derived from *B*. *pseudomallei* [*bapA*TC], a strongly fluorescent signal with a mobility corresponding to approximately 120 kDa was observed (Fig 1A). Although the size of BapA is predicted to be 88 kDa, as no fluorescent signals were observed at this position in any other samples including the *B*. *pseudomallei* [pBHR1] control sample, we concluded that this protein corresponds to TC-tagged BapA. A fluorescent signal corresponding to the predicted size of BapC (23 kDa) was observed in samples derived from the *B*. *pseudomallei* [*bapC*TC] strain (Fig 1A). A fluorescent signal of approximately 25 kDa (Fig 1A) was observed in samples derived from both the [*bopE*TC] and [pBHR1] strains, indicating that some endogenous *B*. *pseudomallei* proteins can form a fluorescent complex with the FlAsH compound, as has been observed previously with this technique [45–47]. These results indicate that the TC-FlAsH^™^ labelling technique can be used for labelling proteins in *B*. *pseudomallei*. No fluorescent signals corresponding to TC-tagged BapB were observed in the *B*. *pseudomallei* [*bapB*TC] samples despite analysis under a wide range of labelling and protein separation conditions (data not shown), suggesting that either BapB is not expressed under the conditions analyzed, or the TC tag was not accessible for FlAsH labelling in the *bapB*TC strain.

**Fig 2:**
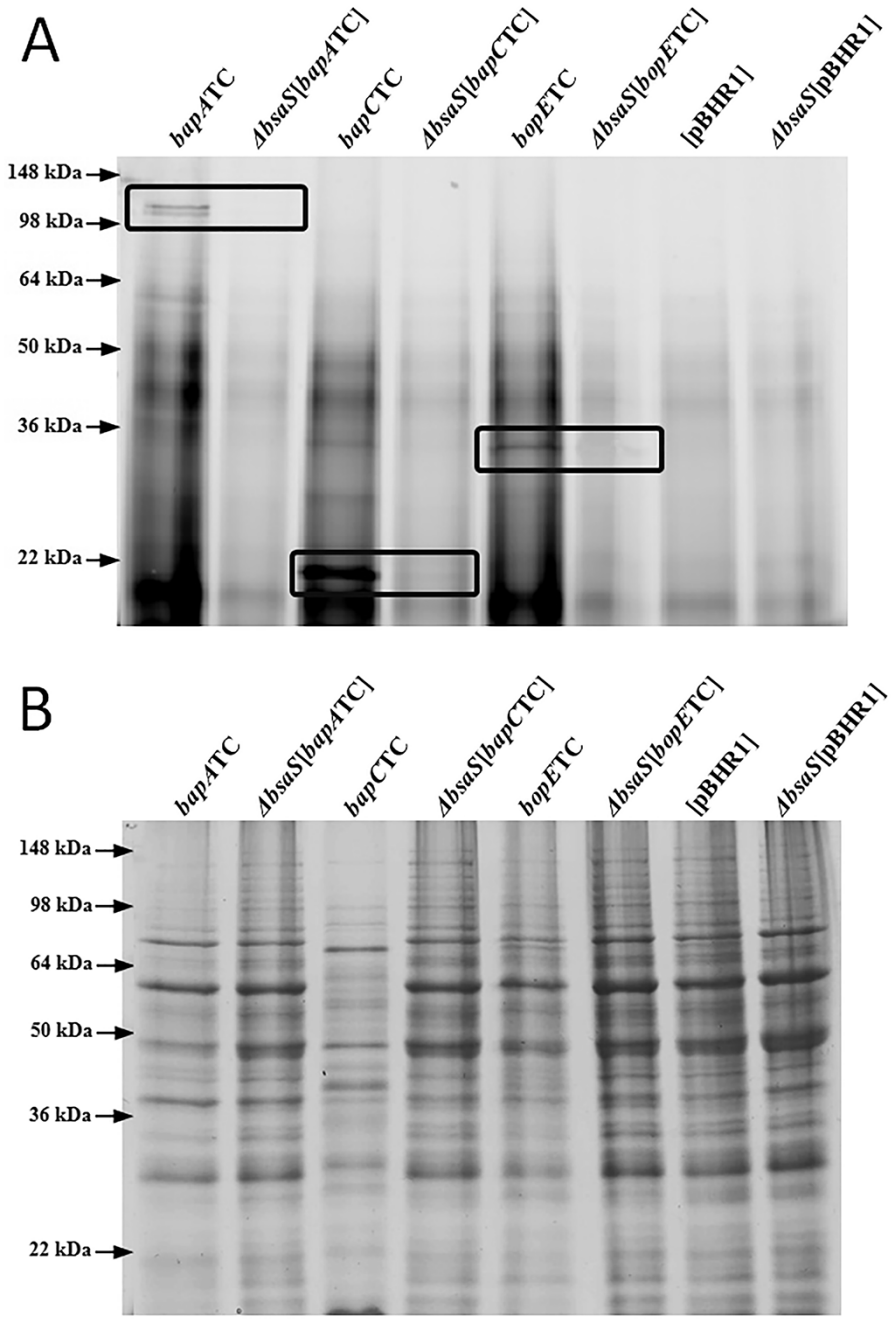
BapA and BapC are secreted in a TTSS3-dependent manner. (A) Proteins in supernatant samples from mid-exponential growth phase cultures of the wild-type ([*bapA*TC], [*bapC*TC], [*bopE*TC] and [pBHR1]) or *ΔbsaS* (*ΔbsaS*[*bapA*TC], *ΔbsaS*[*bapC*TC], *ΔbsaS* [*bopE*TC] and *ΔbsaS*[pBHR1]) strains were precipitated using DOC/TCA and labeled with the FlAsH reagent. (B) Proteins in unlabeled samples were separated by SDS-PAGE and visualized with Coomassie Brilliant Blue staining. Protein bands at or near the expected size of TC-tagged BapA (120 kDa), BapC (23 kDa) and BopE (33 kDa) are indicated by the boxes. The positions of molecular mass markers are shown on the left.

To determine whether BapA and BapC are TTSS3 secreted, we analyzed culture supernatants of the *B*. *pseudomallei* wild-type strain and a TTSS mutant (*bsaS*) strain [30]. Strains were grown to mid-exponential phase, supernatant samples collected, proteins precipitated with DOC/TCA and then labeled with the FlAsH reagent (Fig 2A). Total proteins were also visualized by PAGE and Coomassie Blue staining as a control for loading (Fig 2B). As expected, a fluorescent signal corresponding to TC-tagged BopE was detected in the DOC/TCA precipitated supernatant samples of the *B*. *pseudomallei* [*bopE*TC] strain (Fig 2A), but not in the TTSS3-deficient *ΔbsaS*[*bopE*TC] strain, confirming the TTSS3-dependent secretion of BopE. In the supernatant samples from *B*. *pseudomallei* [*bapA*TC] cultures, a fluorescent signal of approximately 120 kDa was observed (Fig 2A) as previously identified for TC-tagged BapA in whole cell lysates (Fig 1A). Similarly, a fluorescently labeled protein of 23 kDa, corresponding to TC-tagged BapC, was identified in the supernatant samples from the *B*. *pseudomallei* [*bapC*TC] strain (Fig 2A). Importantly, no fluorescently labeled proteins were detected in samples derived from strains *ΔbsaS*[*bapA*TC] or *ΔbsaS*[*bapC*TC] (Fig 2A). These data strongly suggest that BapA and BapC are secreted in a TTSS3-dependent manner.

### BapB plays a role in modulating BopE secretion

**Fig 3:**
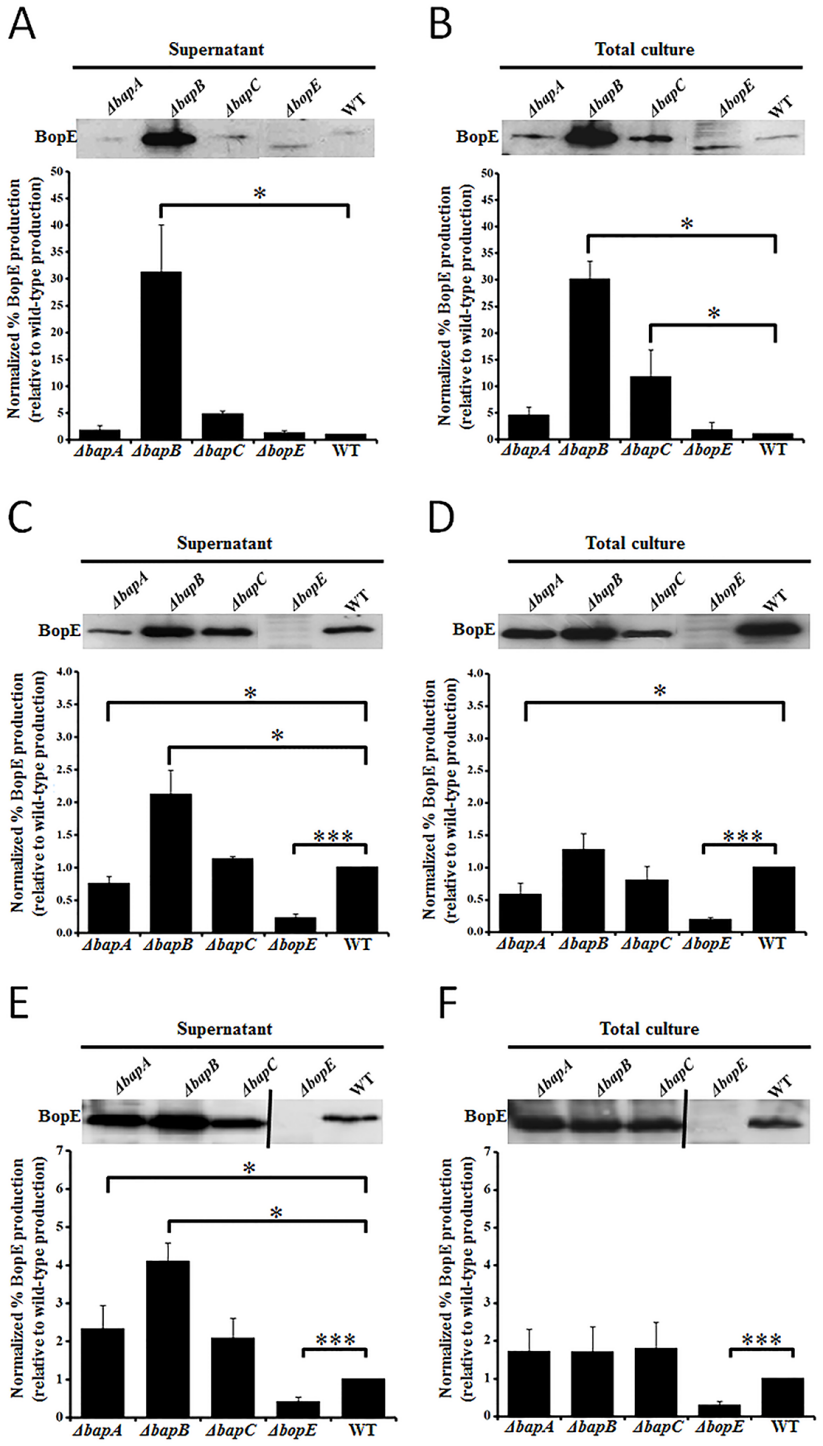
BopE expression and secretion during early- (A, B), mid- (C, D) and late-exponential (E, F) growth phases. Supernatant or total culture samples were separated by SDS-PAGE and transferred to a membrane prior to western immunoblotting using anti-BopE antiserum. The relative expression levels, determined by densitometric analysis of the western blots and normalized against the wild-type expression, are presented as the mean ± SEM of three biological replicates. *P < 0.05, ***P < 0.0001. A single representative western blot is shown above the quantified averaged data for comparison. A vertical black line on the western blot indicates unrelated intervening lanes have been removed. The samples were derived from the *ΔbapA*, *ΔbapB*, *ΔbapC*, *ΔbopE* or the the wild-type (WT) strain.

To determine whether BapA, BapB or BapC are essential for the function or regulation of the TTSS3 we used the secretion of BopE as a measure of TTSS3 activity. The secretion of BopE was assessed with BopE-specific antiserum in each of the *B*. *pseudomallei ΔbapA*, *ΔbapB* and *ΔbapC* strains, and the *B*. *pseudomallei ΔbopE*::pDM4 strain as a negative control. The levels of BopE were assessed in whole cell lysates and culture supernatants collected from strains grown to early-, mid- or late-exponential growth phase (Fig 3).

BopE secretion at levels similar to, or higher than, that observed in the wild-type strain was observed in certain growth phases for all strains except *ΔbopE*::pDM4, indicating that BapA, BapB and BapC are not essential TTSS3 secretion apparatus components. In early-exponential phase culture supernatants (Fig 3A), the level of secreted BopE was indistinguishable in the wild-type, *ΔbapA* and *ΔbapC* strains, but was substantially increased (30-fold; *P* < 0.05) in the *ΔbapB* strain. Similarly, a 30-fold increase in BopE was also observed in total culture samples derived from the *ΔbapB* strain (Fig 3B). Increased BopE production was also observed in total culture samples derived from the *ΔbapC* strain (10-fold; *P* < 0.05) (Fig 3B). Analysis of mid-exponential phase cultures indicated that again the *ΔbapB* strain exhibited a significant increase in secretion of BopE (2-fold, *P* < 0.05). However, no increase was observed in the level of BopE present in the total cultures of the *ΔbapB* strain. In late-exponential phase, the *ΔbapB* strain again showed an increase in secreted BopE (4-fold, *P* < 0.05) (Fig 3E), but no difference was observed in total cultures (Fig 3F). Thus, the *ΔbapB* strain showed a consistent increase in secreted BopE production at all growth phases tested. The *ΔbapA* strain showed a minor decrease in BopE production during mid-exponential phase, both in secreted samples (1.5-fold, *P* < 0.05) (Fig 3C) and total cultures (2-fold, *P* < 0.05) (Fig 3D) and this strain exhibited a slight increase (2-fold; *P* < 0.05) in secreted BopE, during late-exponential phase.

### The transcription of *bopE* is increased in the *ΔbapB* strain

**Fig 4:**
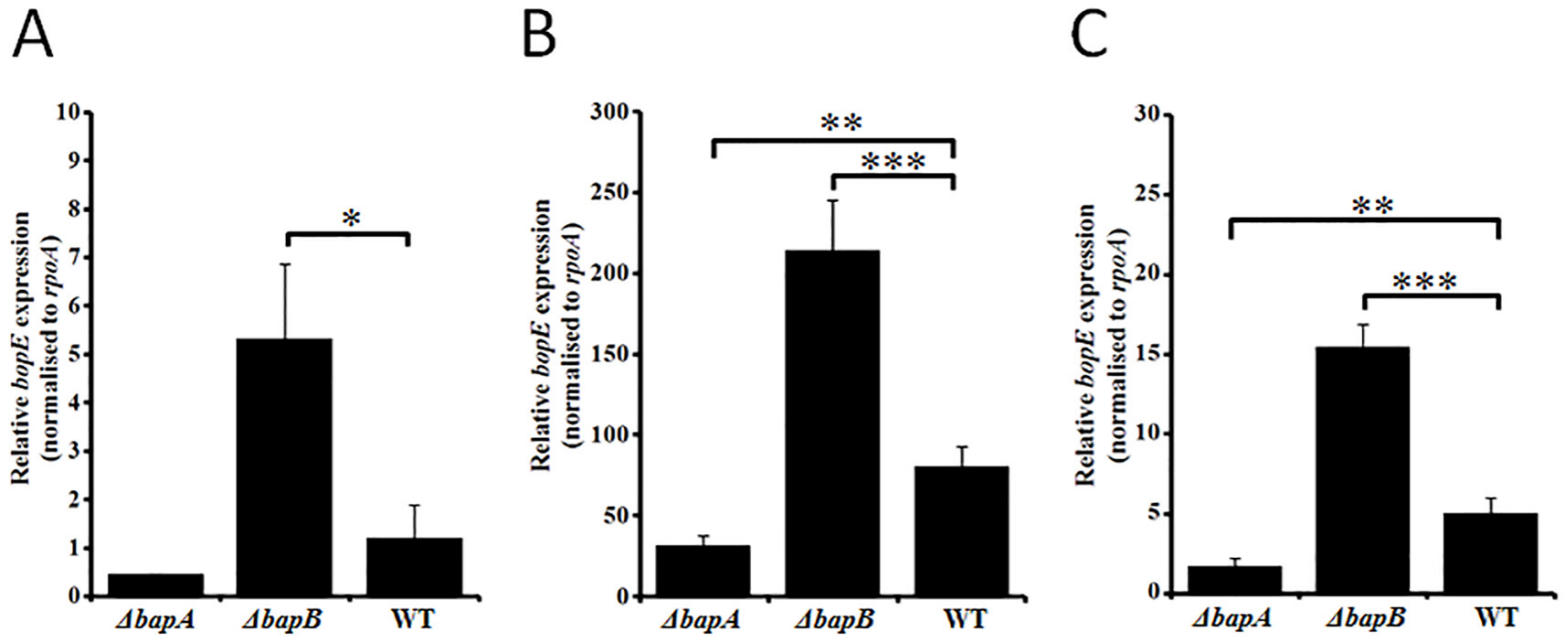
Transcription of *bopE* during the early (A) -, mid (B)—and late (C) -exponential growth phases. The level of *bopE* transcription in each sample was normalized to the expression of the housekeeping gene *rpoA*. The different strains analyzed were: *ΔbapA*, the *ΔbapA* strain; *ΔbapB*, the *ΔbapB* strain; WT, the wild-type strain. Data are expressed as mean ± SEM. Error bars represent the SEM from three technical replicates of biological duplicates. *P < 0.05, **P < 0.001, ***P < 0.0001.

Given the substantial increase in BopE secretion observed in the *ΔbapB* strain across all growth phases, and the increase in total BopE production observed in early-exponential phase total culture samples, we investigated the transcription of *bopE* using quantitative RT-PCR to determine if these changes might be due to increased expression of *bopE*. The transcription of *bopE* was assessed in early-, mid- and late-exponential phase cultures of the *B*. *pseudomallei ΔbapA*, *ΔbapB* and wild-type strains. At early-exponential phase (Fig 4A), the level of expression of *bopE* in the wild-type and *ΔbapA* strains was indistinguishable, but in the *B*. *pseudomallei ΔbapB* strain it was increased approximately 5-fold (*P* < 0.05). At both mid-exponential (3-fold; *P* < 0.0001) and late-exponential (3-fold; *P* < 0.0001) growth phases, *bopE* expression was also significantly increased in the *ΔbapB* strain (Fig 4B). Thus, *bopE* expression was increased between 3- to 5-fold in the *B*. *pseudomallei ΔbapB* strain during early-, mid- and late-exponential growth phases. The expression of *bopE* was reduced in the *ΔbapA* strain at both mid-exponential (3-fold; *P* < 0.001) and late-exponential (4-fold; *P* < 0.001) growth phases.

### BapA, BapB and BapC play a minor role in the *in vivo* fitness of *B*. *pseudomallei*

**Fig 5:**
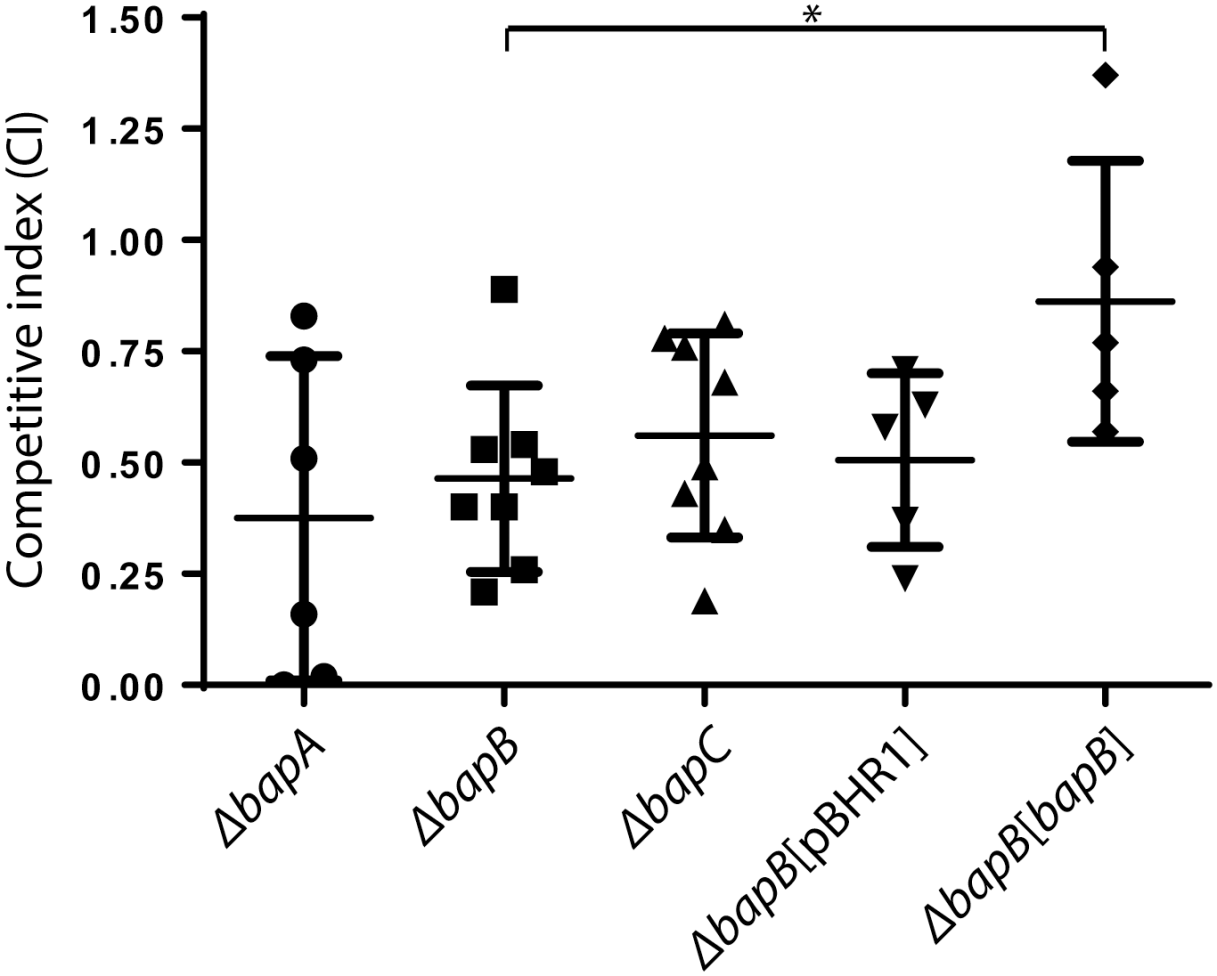
Competitive *in vivo* growth indices for each of the *ΔbapA*, *ΔbapB* and *ΔbapC* mutant strains and the *ΔbapB*[pBHR1] and *ΔbapB*[*bapB*] complemented strains compared to the wild-type strain. Each point on the graph indicates the competitive index (CI) measured in a single mouse. The horizontal marks show the average CI ± 1 SEM. *P < 0.05.

To examine the importance of *bapA*, *bapB* and *bapC* for *in vivo* growth, growth assays were conducted in BALB/c mice and competitive indices (CI) determined. The CI of the *B*. *pseudomallei ΔbapA*, *ΔbapB* and *ΔbapC* strains was significantly reduced compared to the wild-type strain (0.38 ± 0.36, 0.46 ± 0.21 and 0.56 ± 0.23, respectively; Fig 5), while the growth rates of the mutant and wild-type strains were indistinguishable in LB *in vitro* (data not shown). Thus, these data suggest that BapA, BapB and BapC play a minor role in *in vivo* growth of *B*. *pseudomallei*.

To verify that the reduced *in vivo* growth of the mutant strains was due specifically to inactivation of the *bap* genes, each of the mutants was complemented with an intact copy of the relevant gene cloned into the plasmid pBHR1 [34]. The complemented strains and the appropriate controls (S1 Table) were tested for *in vivo* growth using competitive growth assays in BALB/c mice. The *B*. *pseudomallei ΔbapB*[*bapB*] complemented strain was first used in a competitive growth assay with the wild-type strain. The average CI of the *B*. *pseudomallei ΔbapB* strain was 0.46 ± 0.21 (Fig 5) and the average CI of the *B*. *pseudomallei ΔbapB*[pBHR1] vector only strain was not significantly different at 0.51 ± 0.20 (Mann Whitney *U* test; *P* > 0.05; Fig 5). However, the average CI of the *B*. *pseudomallei ΔbapB*[*bapB*] complemented strain was 0.86 ± 0.32, indicating significant, albeit partial, restoration of the wild-type *in vivo* fitness phenotype (Mann Whitney *U* test; *P* = 0.01).

As the *bapB* complementation construct did not fully restore wild-type *in vivo* fitness we assessed the stability of the pBHR1 constructs in the absence of selection for 12 h and 20 h. For the *B*. *pseudomallei ΔbapB*[pBHR1] vector only strain, 64% of cells retained the plasmid after 12 h and 23% after 20 h. For the *ΔbapB*[*bapB*] complemented strain, only 9% of cells retained the plasmid after 20 h. Thus, the partial restoration of *in vivo* growth rate observed for the *B*. *pseudomallei ΔbapB*[*bapB*] strain was obtained despite significant plasmid instability. The rate of plasmid retention for the *B*. *pseudomallei ΔbapC*[*bapC*] and *ΔbapA*[*bapA*] complemented strains was even lower, with no retention of either complementation plasmid observed following 20 h growth in the absence of antibiotic selection (data not shown). Accordingly, we did not test the complemented strains further.

### BapA, BapB and BapC are not essential for bacterial invasion, intracellular survival and replication, escape from host phagosomes, actin-mediated motility or multinucleated giant cell (MNGC) formation

*B*. *pseudomallei* TTSS3 mutants display a range of altered phenotypes, including reduced invasion, intracellular survival and replication, endosomal escape, MNGC formation and intercellular migration [16,19,20,26,27,48]. Therefore, we analyzed the *bapA*, *bapB* and *bapC* mutants with regard to their possible role(s) in bacterial pathogenesis using a range of *in vitro* phenotypic assays, specific for previously known TTSS3 functions [9,20,23,26,27,31,49]. Each of the *B*. *pseudomallei ΔbapA*, *ΔbapB and ΔbapC* strains was able to invade human lung epithelial A549 cells, as efficiently as the wild-type strain (data not shown). In addition, all the *bap* mutant strains retained the ability to survive and multiply within the murine macrophage-like cell line RAW 264.7, escape from host phagosomes, induce MNGC formation and form actin tails for actin-based motility (data not shown), indicating that the *bap* genes are not required for any of these functions.

## Discussion

### BapA and BapC are TTSS3-secreted proteins

The *B*. *pseudomallei* TTSS3 is a critical virulence factor, with the secreted effector proteins directly responsible for the intracellular effects of the TTSS and likely to play key roles in modulating host cell functions [50]. In order to identify novel effector proteins, fluorescent-tagging of putative effectors [51,52] was employed in this study. This technique has been used with success to investigate secretion of TTSS effectors in *S*. *flexneri*, Enteropathogenic *E*. *coli* (EPEC) and *S*. *enterica* serovar Typhimurium [38,41,53]. We fused the BapA, BapB and BapC proteins with a C-terminal TC tag and the secretion of the fusion proteins assessed by FlAsH labelling. As a positive control, the well-characterized TTSS3 effector BopE [27] was TC-tagged and confirmed as being secreted in a TTSS3-dependent manner. Our optimized labeling conditions were then used to investigate the secretion of the TC-tagged BapA, BapB and BapC proteins and show that BapA and BapC were secreted in a TTSS3-dependent manner. To our knowledge, this is the first time FlAsH labelling has been used to investigate the secretion of *B*. *pseudomallei* TTSS molecules. Very recently, BapA was shown to be a *bone fide* secreted TTSS3 effector, by initial identification of secreted proteins in a *B*. *pseudomallei* hypersecretion mutant and then by using a c-Myc epitope tagging strategy [32]; our data confirm this result. Furthermore, we identified BapC as a novel TTSS3-secreted protein. Interestingly, BapC was not identified as a T3SS3 effector via proteomic analysis of *B*. *pseudomallei* hypersecretion mutants [32]. It is possible that BapC secretion is not significantly increased in the *bsaP* or *bipD* mutants used in that study or that even in hypersecreting strains the total amount of secreted BapC is below detection levels. However, with the sensitive detection of FlAsH labeling, our analysis was able to identify BapC as a *bone fide* TTSS3 effector using wild-type *B*. *pseudomallei*. In our hands, the TC-tagged BapB could not be identified either in total cultures or supernatant samples. As we have shown by RT-PCR that *bapB* is expressed during *in vitro* growth (data not shown), the lack of labelling suggests that the TC tag in BapB was not accessible for FlAsH compound binding. Based on bioinformatics analyses, BapB is a predicted acyl carrier protein (ACP) containing a pantetheine 4' phosphate (PP) group. The PP prosthetic group has high conformational flexibility and functions in the transfer of acyl molecules during fatty acid biosynthesis [54,55]. Given that the distance between the two pairs of cysteines in the TC fusion protein has to be accurately matched to the spacing of the bi-arsenic molecules of the FlAsH compound [56], such conformational flexibility may inhibit the TC-FlAsH interaction [46].

### BapB affects secretion of BopE

To test whether loss of BapA, BapB or BapC affected the activity of the TTSS3, production and secretion of the known effector BopE were used as markers of TTSS3 activity. All mutant strains were able to secrete BopE, showing that BapA, BapB and BapC are not essential for TTSS3 function. However, the level of secreted BopE was significantly increased in the *B*. *pseudomallei ΔbapB* strain at all three growth phases tested, and total production of BopE was also significantly higher in the *ΔbapB* strain at early-exponential growth phase. Furthermore, there was significantly increased transcription of the *bopE* gene in the *ΔbapB* strain at all growth phases tested. These data suggest that BapB levels negatively affect the transcription of *bopE*. A similar scenario has been found in an AraC-family TTSS regulator BsaN (BPSS1546), which in association with its chaperone BicA (BPSS1533), functions by controlling the expression of not only several TTSS3 components, including BopA, BopE, BapA, BapB and BapC, but also T6SS molecules involved in facilitating bacterial survival within the host, thereby promoting bacterial virulence [57].

As stated above, BapB is a predicted ACP that displays most similarity to the TTSS ACP IacP of *S*. *enterica* serovar Typhimurium (27% amino acid identity). A *Salmonella iacP* mutant demonstrated reduced invasion of non-phagocytic cells, altered host actin rearrangement, a significant decrease in secretion of the effector proteins SopA, SopB and SopC and increased secretion of the flagella subunit FljB [58]. By contrast, the *ΔbapB* strain showed no difference in invasion of A549 cells (data not shown) and increased effector secretion (Fig 3); thus, IacP and BapB are unlikely to have similar functions. While BapB shows highest amino acid identity to IacP, other features of BapB, such as its acidic pI and small size, suggest it may function as a TTSS3 chaperone. In general, TTSS chaperones are essential not only for preventing the degradation and/or misfolding of bacterial effector proteins prior to secretion into the host, but also play important roles in preventing undesirable interactions of the effectors with other TTSS components [59–62]. TTSS chaperones can broadly be divided into different groups (class IA, IB, II and III) according to their substrate specificities [63]. Phylogenetic analysis of BapB and other TTSS chaperone proteins from *Yersinia*, *Shigella* and *Salmonella* spp. (S1 Fig) indicated that BapB clustered with the class III chaperone FliT. Among the class III chaperones, FliT functions as a negative regulator of the flagella biosynthesis operon of *Salmonella* [64–66]. Moreover, some TTSS chaperones, such as *Salmonella* SicA and *Shigella* IpgC play a role in regulating the transcription of effector genes [63,67,68,69]. Taken together, these data suggest a possible function of BapB as a TTSS3 chaperone with a negative regulatory function. Although BapB was not identified by a large scale bioinformatics genomic screen to identify putative TTSS chaperone-effector pairs in bacterial genomes, this may be because that screen applied a molecular weight filter of between 12 and 20 KDa and BapB is slightly smaller than this at 10 KDa [70].

The secretion of BopE in the *ΔbapA* strain was similar to that of the wild-type strain at early-exponential growth phase, but showed a slight decrease and then increase at mid and late-exponential growth phase, respectively. Surprisingly, transcription of *bopE* in the *ΔbapA* strain was slightly decreased at both mid and late-exponential growth phases. These data clearly show that BapA is not required for the TTSS3 function, but indicate that loss of BapA may play a minor or indirect role in regulation of TTSS activity.

BapC is predicted to contain a TTSS-associated lytic transglycosylase (LT) domain. In general, proteins with LT activity function by cleaving the β-1,4 glycosidic bond between *N*-acetylmuramoyl and *N*-acetylglucosaminyl residues of bacterial peptidoglycan (PG) for the recycling of PG, cell division and insertion of either flagella or secretion system components including the TTSS [71–74] into the membrane. Alignment of the amino acid sequence of BapC with other TTSS proteins that contain LT domains indicated that the two TTSS-associated LTs, HpaH and Hpa2, from the plant pathogens *Xanthomonas campestris* pv. vesicatoria and *X*. *oryzae* pv. oryzae, respectively, displayed the highest identity (approximately 39%; S2 Fig). HpaH plays a role in promoting TTSS assembly and secretion of other TTSS proteins, at least in part, by remodeling PG [75]. However, the secretion of HpaH, as a TTSS effector, has not yet been verified. The *X*. *oryzae* Hpa2 is likely to function as part of the translocon complex and together with another translocon component HrpF interacts with the host cell membrane [76,77]. Both HpaH and Hpa2 are required for bacterial virulence [75–77]. BapC also shows identity (approximately 36%) with the putative TTSS effectors, IagB and IpgF from *Salmonella* and *Shigella* respectively. Both these proteins share the three conserved motifs of the LT domain with BapC (S2 Fig) and cleave PG. However, IagB and IpgF play roles in bacterial invasion, but not in virulence [78–80]. Thus, the role of BapC in *B*. *pseudomallei* TTSS3 function appears different from each of these related TTSS proteins.

Taken together, these data suggest that BapA, BapB and BapC act to modulate the efficient function of the TTSS3 needle-like apparatus. None of the proteins is essential for TTSS function, but production of BapA, BapB and BapC appears necessary for full *B*. *pseudomallei in vivo* fitness in the BALB/c mouse model. BapA and BapC are clearly TTSS3 secreted proteins and BapC may play a role in facilitating TTSS needle protrusion and expansion through the PG. BapB may act both as a chaperone and a negative regulator that prevents premature secretion of certain TTSS3 effectors.

## Supporting Information

S1 Fig Phylogenetic tree analysis of representative different classes of TTSS chaperones [63] based on ClustalW alignment (www.genome.jp/tools/clustalw). BapB is highlighted in a black box.
(TIF)

S2 Fig Amino acid sequence alignment of *B*. *pseudomallei* BapC, *X*. *campestris* pv. vesicatoria HpaH, *X*. *oryzae* pv. oryzae Hpa2, *Salmonella* IagB and *Shigella* IpgF. The three conserved motifs of the LT domains are indicated by the boxes. Regions 1 and 3 are predicted to form α-helices and region 2 is predicted to form a β-sheet. Residue E in each of the α-helix regions (boxed) is typically the catalytic glutamate residue responsible for LT domain cleavage of β-1,4 glycosidic bonds of bacterial peptidoglycan. ‘:’ or ‘.’ designate amino acids with strongly and weakly conserved properties, respectively. ‘*’ designates identical amino acids.
(TIF)

S1 Table Strains and plasmids used in this study. (DOCX)

S2 Table Primers used in this study. (DOCX)

## Abbreviations

ACP: acyl carrier protein
Bap: *Burkholderia* associated protein
CI: competitive index
GFP: green fluorescent protein
LC3: microtubule-associated protein light chain 3
LPS: lipopolysaccharide
LT: lytic transglycosylase
MNGC: multinucleated giant cell
TC: tetracysteine
TTSS: type III secretion system
