# Supplementary figures and images for: The Burkholderia pseudomallei Proteins BapA and BapC Are Secreted TTSS3 Effectors and BapB Levels Modulate Expression of BopE
Source: PLoS One. 2015 Dec 1;10(12):e0143916. doi: 10.1371/journal.pone.0143916 (PMC4666416; doi:10.1371/journal.pone.0143916)

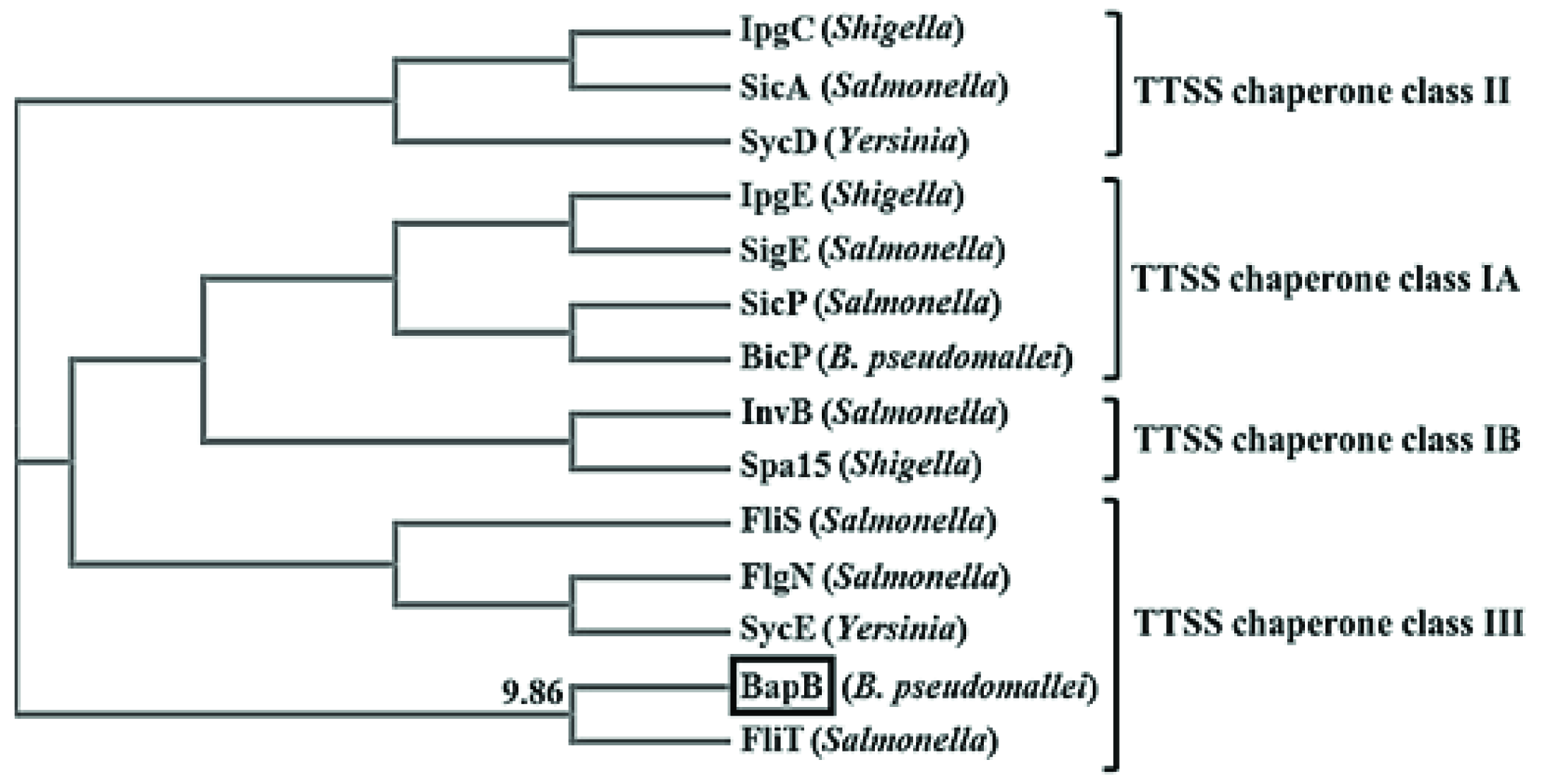

Supplement: S1 Fig — BapB is highlighted in a black box. (TIF) [file pone.0143916.s001.tif]

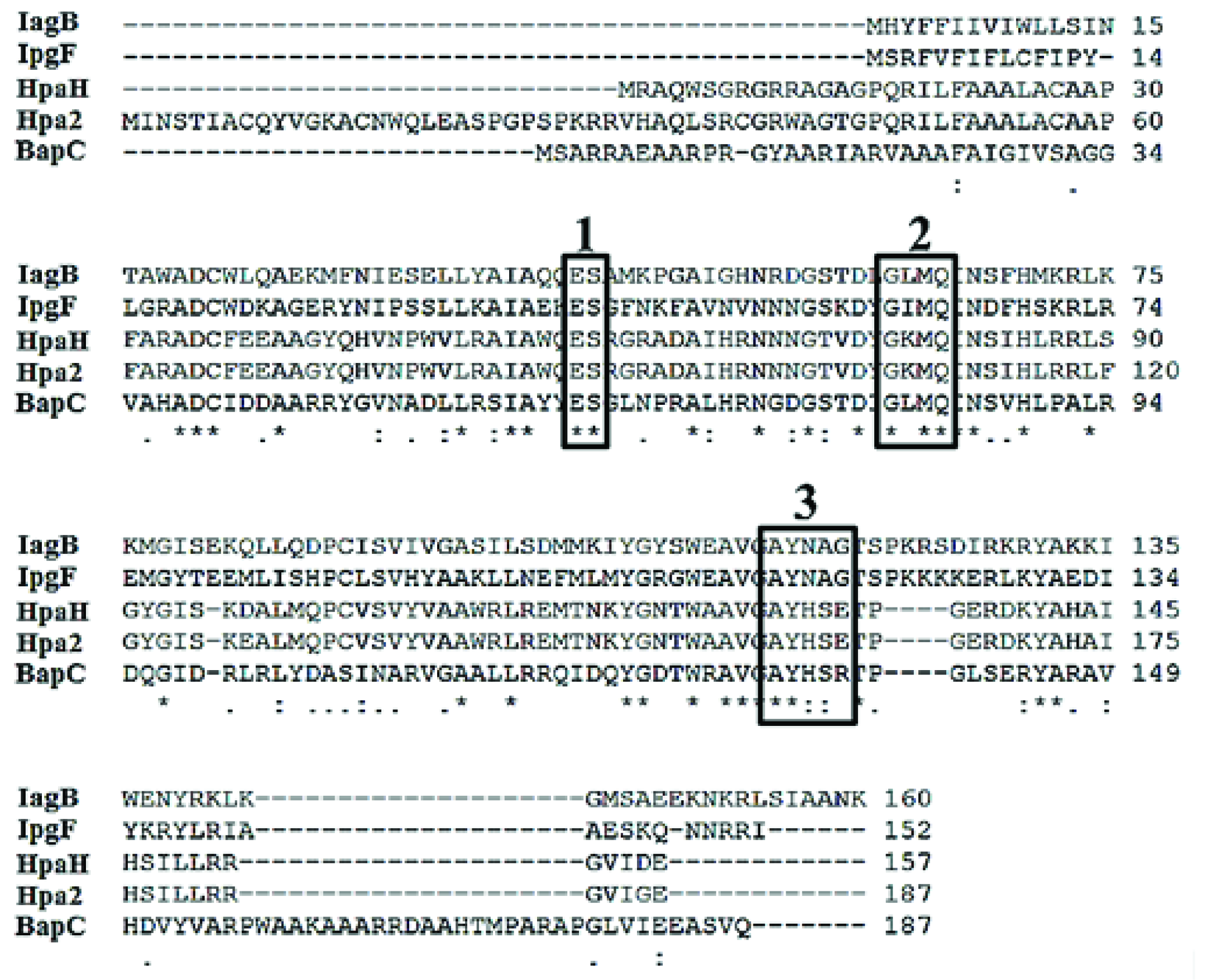

Supplement: S2 Fig — The three conserved motifs of the LT domains are indicated by the boxes. Regions 1 and 3 are predicted to form α-helices and region 2 is predicted to form a β-sheet. Residue E in each of the α-helix regions (boxed) is typically the catalytic glutamate residue responsible for LT domain cleavage of β-1,4 glycosidic bonds of bacterial peptidoglycan. ‘:’ or ‘.’ designate amino acids with strongly and weakly conserved properties, respectively. ‘*’ designates identical amino acids. (TIF) [file pone.0143916.s002.tif]
