# Supplementary material for: The Burkholderia pseudomallei Proteins BapA and BapC Are Secreted TTSS3 Effectors and BapB Levels Modulate Expression of BopE
Source: PLoS One. 2015 Dec 1;10(12):e0143916. doi: 10.1371/journal.pone.0143916 (PMC4666416; doi:10.1371/journal.pone.0143916)
Supplement: S1 Table — (DOCX) [file pone.0143916.s003.docx]

| **Strains or plasmids** | **Genotype and/or phenotype** | **Reference** |
| --- | --- | --- |
| ***B. pseudomallei*** |  |  |
| K96243 | Wild-type, clinical isolate from Thailand | [[32](#_ENREF_32)] |
| *ΔbapA* | \| K96243 derivative with a 1,565-bp deletion within *bapA* replaced by a 1.3-kb  tetracycline resistance cassette derived from pUTminiTn*5*Tc, Tet^R^ \| \| --- \| | This study |
| *ΔbapB* | K96243 derivative with a central fragment of *bapB* replaced by a 1.3-kb tetracycline resistance cassette from pUTminiTn5Tc, Tet^R^ | This study |
| *ΔbapC* | K96243 derivative with a central fragment of *bapC* replaced by a 1.3-kb tetracycline resistance cassette from pUTminiTn*5*Tc, Tet^R^ | This study |
| *ΔbapA*[*bapA*] | The *ΔbapA* strain complemented with the pBHR1::*bapA* construct, Tet^R^, Kan^R^ | This study |
| *ΔbapB*[*bapB*] | The *ΔbapB* strain complemented with the pBHR1::*bapB* construct, Tet^R^, Kan^R^ | This study |
| *ΔbapC*[*bapC*] | The *ΔbapC* strain complemented with the pBHR1::*bapC* construct, Tet^R^, Kan^R^ | This study |
| *ΔbapA*[pBHR1] | The *ΔbapA* strain harbouring empty pBHR1 vector, Tet^R^, Kan^R^, Cm^R^ | This study |
| *ΔbapB*[pBHR1] | The *ΔbapB* strain harbouring empty pBHR1 vector, Tet^R^, Kan^R^, Cm^R^ | This study |
| *ΔbapC*[pBHR1] | The *ΔbapC* strain harbouring empty pBHR1 vector, Tet^R^, Kan^R^, Cm^R^ | This study |
| K96243[pBHR1] | K96243 derivative harbouring empty pBHR1 vector, Kan^R^, Cm^R^ | This study |
| *bopE*TC | K96243 derivative with a tetracysteine (TC) tagged *bopE*. Insertion of the pUC18Tmini-Tn*7*T::*tetA*(C)::*P_glmS2_*::*bopE*TC construct into the bacterial genome, Tet^R^ | This study |
| K96243[*bopE*TC] | K96243 derivative harbouring the pBHR1::*P_glmS2_*::*bopE*TC construct, Kan^R^ | This study |
| K96243[*bapA*TC] | K96243 derivative harbouring the pBHR1::*bapA*TC construct, Kan^R^ | This study |
| K96243[*bapB*TC] | K96243 derivative harbouring the pBHR1::*bapB*TC construct, Kan^R^ | This study |
| K96243[*bapC*TC] | K96243 derivative harbouring the pBHR1::*bapC*TC construct, Kan^R^ | This study |
| *ΔbsaS* | K96243 derivative with a central fragment of *bsaS* replaced by a 1.3-kb tetracycline resistance cassette derived from pUTminiTn*5*Tc, Tet^R^ | [[30](#_ENREF_30)] |
| *ΔbsaS*[*bopE*TC] | *ΔbsaS* derivative harboring the pBHR1::*P_glmS2_*::*bopE*TC::T_1_T_0_ construct, Tet^R^, Kan^R^ | This study |
| *ΔbsaS*[*bapA*TC] | *ΔbsaS* derivative harboring the pBHR1::*bapA*TC construct, Tet^R^, Kan^R^ | This study |
| *ΔbsaS*[*bapB*TC] | *ΔbsaS* derivative harboring the pBHR1::*bapB*TC construct, Tet^R^, Kan^R^ | This study |
| *ΔbsaS*[*bapC*TC] | *ΔbsaS* derivative harboring the pBHR1::*bapC*TC construct, Tet^R^, Kan^R^ | This study |
| *ΔbsaS*[pBHR1] | *ΔbsaS* derivative harboring empty pBHR1 vector, Tet^R^, Kan^R^, Cm^R^ | This study |
| *ΔbopE*::pDM4 | K96243 derivative harboring the pDM4::*bopE* construct, Cm^R^ | This study |
| ***E. coli*** |  |  |
| DH5α | General *E. coli* strain used for plasmid amplification, transformation and storage: *F^–^, ø80dlacZΔM15, Δ(lacZYA-argF)U169, recA1, endA1, hsdR17(rK^–^, mK^+^), phoA, supE44, λ–, thi-1, deoR*, *gyrA96*, *relA1* | [[78](#_ENREF_78)] |
| S17-1/λ*pir* | Strain for propagation of pDM4 and pBHR1, contains RP4 transfer genes integrated into the chromosome: *recA, thi, pro, hsdR-M^+^* (F^−^) RP4-2-Tc::Mu *aphA*::Tn*7* λ*pir* lysogen Tp^R^, Sm^R^ | [[79](#_ENREF_79)] |
| **Plasmids** |  |  |
| pDM4 | λ*pir*-dependent replication, suicide vector in *B. pseudomallei*, *oriR6K*, *mobRP4*, *sacBR*, Cm^R^ | [[34](#_ENREF_34)] |
| pDM4::*bapA*::*tetA*(C) | pDM4 containing a 918-bp fragment harbouring the 5’ region of *bapA*, tetracycline resistance cassette and a 1,241-bp fragment encompassing the 3’ region of *bapA* and the entire *bapB* and *bapC* genes, Cm^R^, Tet^R^ | This study |
| pDM4::*bapB*::*tetA*(C) | pDM4 containing a 1,093-bp fragment encompassing the 3’ region of *bapA* and the 5' region of *bapB*, tetracycline resistance cassette and a 662-bp fragment containing the 3' region of *bapB* and the entire *bapC* genes, Cm^R^, Tet^R^ | This study |
| pDM4::*bapC*::*tetA*(C) | pDM4 containing a 753-bp fragment encompassing the 3’ region of *bapA*, the entire *bapB* and the 5' region of *bapC*, tetracycline resistance cassette and a 755-bp fragment containing the 3’ region of *bapC* and the downstream region, Cm^R^, Tet^R^ | This study |
| pDM4::*bopE* | pDM4 containing a 367-bp internal fragment of *bopE* derived from *B. pseudomallei* strain 10276 for generating the *bopE* single cross-over mutant, Cm^R^ | [[26](#_ENREF_26)] |
| pUTminiTn*5*Tc | Mini-Tn*5*Tc in plasmid pUT, Amp^R^, Tet^R^ | [[35](#_ENREF_35)] |
| pUC18Tmini-Tn*7*T:: *tetA*(C)::*P_glmS2_* | pUC18Tmini-Tn*7*T containing tetracycline resistance cassette derived from pUTminiTn*5*Tc and *glmS2* promoter, Amp^R^, Tet^R^ | This study |
| pUC18Tmini-Tn*7*T::  *tetA*(C)::*P_glmS2_*::*bopE*TC | pUC18Tmini-Tn*7*T containing tetracycline resistance cassette derived from pUTminiTn*5*Tc, *glmS2* promoter and full length *bopE* derived from *B. pseudomallei* strain K96243 tagged with the TC motif, Amp^R^, Tet^R^ | This study |
| pBHR1 | A mobilisable *E. coli/B. pseudomallei* shuttle plasmid. Cm^R^, Kan^R^ | [[28](#_ENREF_28),[33](#_ENREF_33)] |
| pBHR1::*bapA* | pBHR1 containing full length *bapA* derived from *B. pseudomallei* strain K96243, Kan^R^ | This study |
| pBHR1::*bapB* | pBHR1 containing full length *bapB* derived from *B. pseudomallei* strain K96243, Kan^R^ | This study |
| pBHR1::*bapC* | pBHR1 containing full length *bapC* derived from *B. pseudomallei* strain K96243, Kan^R^ | This study |
| pBHR1::*bapA*TC | pBHR1 containing full length *bapA*, derived from *B. pseudomallei* strain K96243, tagged with the TC motif, Kan^R^ | This study |
| pBHR1::*bapB*TC | pBHR1 containing full length *bapB*, derived from *B. pseudomallei* strain K96243, tagged with the TC motif, Kan^R^ | This study |
| pBHR1::*bapC*TC | pBHR1 containing full length *bapC*, derived from *B. pseudomallei* strain K96243, tagged with the TC motif, Kan^R^ | This study |
| pBHR1::*P_glmS2_*::*bopE*TC | pBHR1 containing a 1,411-bp fragment, derived from *B. pseudomallei* strain *bopE*TC, harboring *glmS2* promoter, a full length version of *bopE* fused with the TC motif and containing two downstream terminators, Kan^R^ | This study |
