# Supplementary material for: The Burkholderia pseudomallei Proteins BapA and BapC Are Secreted TTSS3 Effectors and BapB Levels Modulate Expression of BopE
Source: PLoS One. 2015 Dec 1;10(12):e0143916. doi: 10.1371/journal.pone.0143916 (PMC4666416; doi:10.1371/journal.pone.0143916)
Supplement: S2 Table — (DOCX) [file pone.0143916.s004.docx]

| **Primer name** | **Sequence (5’ – 3’)*** | **Note** |
| --- | --- | --- |
| MC5516 | GGGCCCAGATCTCTTTATCCGCTCGTCGACGATGCTT | Forward primer for amplification of the 3' region of *bapA* and the entire *bapB* and *bapC* genes, specifying a *Bgl*II site |
| MC5517 | GGGCCCTCTAGATTGGCGTATTGGCGTATTGGCGTA | Reverse primer for amplification of the 3' region of *bapA* and the entire *bapB* and *bapC* genes, specifying an *Xba*I site |
| MC5532 | GGGCCCACTAGTCCGATCCGAAGCAACCGACAAGA | Forward primer for amplification of the 5' region of *bapA*, specifying a *Spe*I site |
| MC5533 | GGGCCCAGATCTACCATGTCGACGAGATTCGTC | Reverse primer for amplification of the 5' region of *bapA*, specifying a *Bgl*II site |
| PA6067 | AGTAGGACAAATCCGCCGCT | Forward primer downstream of the multiple cloning site of pUC18T mini-Tn*7*T |
| PA6068 | ATCTGGTTGGCCTGCAAGGC | Reverse primer downstream of the multiple cloning site of pUC18T mini-Tn*7*T MCS |
| JT6073 | ATGCGGGTGATGCGGGTGAT | Forward primer upstream of *bapB* |
| JT6079 | CGGTATGTGGCTTCGAGCGT | Forward primer for amplification of *bopE*-containing fragment |
| JT6080 | CGAAACGCTCGGGCAACTGT | Reverse primer for amplification of *bopE*-containing fragment |
| JT6156 | CGGCTGACGCAATCGCG | Forward primer for amplification of the 1,093-bp 3' region of *bapA* and the 5' region of *bapB*, containing a native *Sph*I site |
| JT6157 | ATCCATCGCCAGGTCGTCGA | Reverse primer for amplification of the 1,093-bp 3' region of *bapA* and the 5' region of *bapB*, containing a native *Sph*I site |
| JT6175 | GGGCCCACTAGTCAGATCGCGCCGCCGCA | Forward primer for amplification of the 662-bp 3' region of *bapB* and the entire *bapC* gene, specifying a *Spe*I site |
| JT6176 | GGGCCCACTAGTGGGCCGCGCGACATAGA | Reverse primer for amplification of the 662-bp 3' region of *bapB* and the entire *bapC* gene, specifying a *Spe*I site |
| JT6179 | TATTACGAGTCGGGGCTGAATCCGCGC | Forward primer for amplification of the 775-bp 3' downstream region of *bapC*, containing a native *Sal*I site |
| JT6180 | GGGCCCGTCGACGGATCGGTGAATTCGTGGGGTTCTCG | Reverse primer for amplification of the 775-bp 3' downstream region of *bapC*, specifying a *Sal*I site |
| JT6319 | GGGCCCCCCGGGGTCACATCGAACGTCGCATC | Forward primer for amplification of the 753-bp 3' region of *bapA*, the entire *bapB* and the 5' region of *bapC*, specifying an *Xma*I site |
| JT6320 | GGGCCCGAGCTCCCCGCCGATACGATGCCGAT | Reverse primer for amplification of the 753-bp 3' region of *bapA*, the entire *bapB* and the 5' region of *bapC*, specifying a *Sac*I site |
| JT6321 | GGGCCCCCCGGGACGATGCCGATCGCGAACGC | Reverse primer at the 5’ end of *bapC*, specifying an *Xma*I site |
| JT6640 | GGGCCCAGATCTGATGGATTCGCTCGAGCTGA | Forward primer in the middle of *bapB*, specifying a *Bgl*II site |
| JT6929 | GGGCCCCCCGGGGACCTCCTTCCCTTCAACCGA | Forward primer for amplification of *bopE*, specifying an *Xma*I site |
| JT6930 | GGGCCCACTAGTCGCGCCGTCCGCCGCGTTCGT | Reverse primer for amplification of *bopE*, specifying an *Spe*I site |
| JT6931 | CTAGTGCGGGCAGCTTCCTGAACTGCTGCCCGGGCTGCTGCATGGAGCCGGGCGGCCGCTAA | Forward primer for generating the TC motif, and for cloning at an *Spe*I site |
| JT6932 | CTAGTTAGCGGCCGCCCGGCTCCATGCAGCAGCCCGGGCAGCAGTTCAGGAAGCTGCCCGCA | Reverse primer for generating the TC motif, and for cloning at an *Spe*I site |
| JT7065 | GGGCCCGAATTCCCCCGATCCGAAGCAACCGACAAG | Forward primer used for amplification of the full length *bapA* including the predicted native promoter, specifying an *Eco*RI site |
| JT7066 | GGGCCCAACGTTTCGCTTCGTGCCGTTGGCGATCGAATC | Reverse primer used for amplification of the full length *bapA* including the predicted native promoter, specifying an *Acl*I site |
| JT7080 | GGGCCCATAACTGCCTTAAAAAAATTA | Forward primer for amplification of the chloramphenicol resistance gene of pBHR1 |
| JT7081 | GGGCCCGTATTTTTTGAGTTATCGAGAT | Reverse primer for amplification of the chloramphenicol resistance gene of pBHR1 |
| JT7125 | GGGCCCAACGTTTAGCAATTTAACTGGTACCG | Forward primer for amplification of the fragment containing the *glmS2* promoter, *bopE* tagged with TC motif and two terminators, from the mini-Tn*7* construct, specifying an *Acl*I site |
| JT7126 | GGGCCCCCATGGATCGATAAGCTAGCTTAATT | Reverse primer for amplification of the fragment containing the *glmS2* promoter, *bopE* tagged with TC motif and two terminators, from the mini-Tn*7* construct, specifying an *Nco*I site |
| JT7147 | CGTTGCGGGCAGCTTCCTGAACTGCTGCCCGGGCTGCTGCATGGAGCCGGGCGGCCGCTAAAA | Forward primer for generating the TC tag, and for cloning at an *Acl*I site |
| JT7171 | GGGCCCGAATTCCGGCACGAAGCGATGACGGCCGGCC | Forward primer used for amplification of the full length *bapB*, specifying an *Eco*RI site |
| JT7172 | GGGCCCAACGTTTGCGCCTCCCGAATCGTCCG | Reverse primer used for amplification of the full length *bapB*, specifying an *Acl*I site |
| JT7173 | GGGCCCGAATTCGGCCGCGCGCGCGGACGATTCGGGAGG | Forward primer used for amplification of the full length *bapC*, specifying an *Eco*RI site |
| JT7174 | GGGCCCAACGTTCTGCACCGACGCCTCCTCGATCACGAGCCC | Reverse primer used for amplification of the full length *bapC*, specifying an *Acl*I site |
| JT7241 | CGTTTTAGCGGCCGCCCGGCTCCATGCAGCAGCCCGGGCAGCAGTTCAGGAAGCTGCCCGCAA | Reverse primer for generating the TC tag, and for cloning at an *Acl*I site |
| JT7472 | TGACTTACAACCCGAGAATCG | Forward primer specific for amplification of *bopE* for qRT-PCR |
| JT7473 | GATGCGCTTGATCTGTTGTG | Reverse primer specific for amplification of *bopE* for qRT-PCR |
| JT7474 | CACGATTGCGAAGTCATCAA | Forward primer specific for amplification of *rpoA* for qRT-PCR |
| JT7475 | GCCCTTTTCCACCTTGATCT | Reverse primer specific for amplification of *rpoA* for qRT-PCR |

*Underlined sequences indicate added restriction sites.
